# Disulfide-Cross-Linked Tetra-PEG Gels

**DOI:** 10.1021/acs.macromol.3c02514

**Published:** 2024-03-25

**Authors:** Zhao Meng, Lucas Löser, Kay Saalwächter, Urs Gasser, Harm-Anton Klok

**Affiliations:** † Institut des Matériaux and Institut des Sciences et Ingénierie Chimiques, Laboratoire des Polymères, École Polytechnique Fédérale de Lausanne (EPFL), Bâtiment MXD, Station 12, CH-1015 Lausanne, Switzerland; ‡ Swiss National Center for Competence in Research (NCCR) Bio-inspired Materials, University of Fribourg, Chemin des Verdiers 4, CH-1700 Fribourg, Switzerland; § Institut für Physik - NMR, Martin-Luther Universität Halle-Wittenberg, Betty-Heimann-Str. 7, 06120 Halle (Saale), Germany; ∥ Laboratory for Neutron Scattering and Imaging (LNS), Paul Scherrer Institut, CH-5232 Villigen PSI, Switzerland

## Abstract

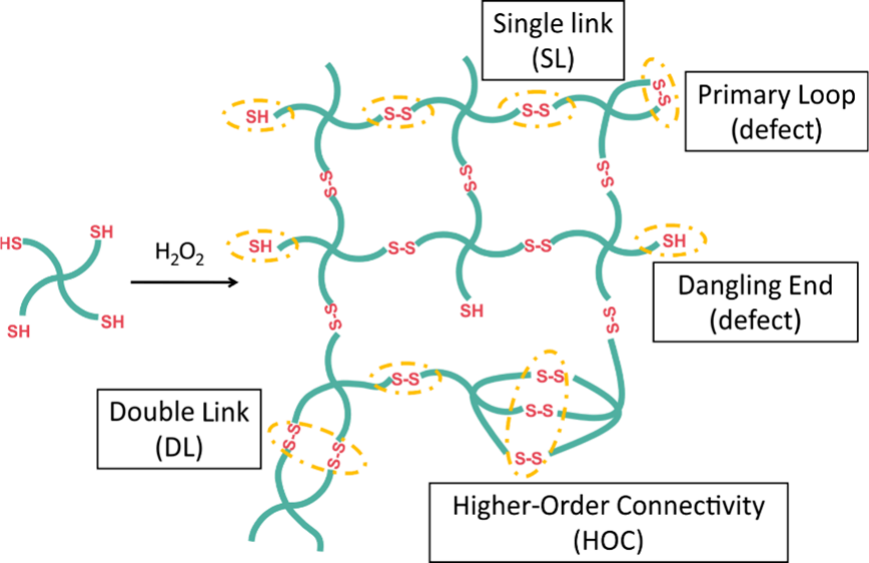

The preparation of polymer gels via cross-linking of four-arm star-shaped poly(ethylene glycol) (Tetra-PEG) precursors is an attractive strategy to prepare networks with relatively well-defined topologies. Typically, Tetra-PEG gels are obtained by cross-linking heterocomplementary reactive Tetra-PEG precursors. This study, in contrast, explores the cross-linking of self-reactive, thiol-end functional Tetra-PEG macromers to form disulfide-cross-linked gels. The structure of the disulfide-cross-linked Tetra-PEG gels was studied with multiple-quantum NMR (MQ-NMR) spectroscopy and small-angle neutron scattering (SANS) experiments. In line with earlier simulation studies, these experiments showed a strong dependence of the relative fractions of the different network connectivities on the concentration of the thiol-end functional Tetra-PEG macromer that was used for the synthesis of the networks. Disulfide-cross-linked Tetra-PEG gels prepared at macromer concentrations below the overlap concentration (*c* = 0.66c*) primarily feature defect connectivity motifs, such as primary loops and dangling ends. For networks prepared at macromer concentrations above the overlap concentration, the fraction of single-link connectivities was found to be similar to that in amide-cross-linked Tetra-PEG gels obtained by heterocomplementary cross-linking of *N*-hydroxysuccinimide ester and amine functional Tetra-PEG macromers. Since disulfide bonds are susceptible to reductive cleavage, these disulfide-cross-linked gels are of interest, e.g., as reduction-sensitive hydrogels for a variety of biomedical applications.

## Introduction

Polymer gels are solvent-swollen polymer networks, which are widely used, e.g., as superabsorbers,^1,2^ contact lens materials,^3^ scaffolds for tissue repair and regeneration,^4−6^ and depots for controlled drug release.^7,8^ Solvent-swollen polymer networks are typically characterized by structural inhomogeneities that span across various length scales.^9−13^ These inhomogeneities include topological defects (such as dangling ends and loops) as well as nanostructured heterogeneities characterized by correlation lengths of 10–100 nm that are due to an inhomogeneous spatial distribution of cross-link density. It is well-established that these structural heterogeneities influence the swelling behavior and macroscopic properties, such as mechanical strength and permeability of polymer gels. As a consequence, there is considerable interest in approaches that allow the preparation of polymer gels with well-defined, including, but not limited to, defect-free, network topologies. The ability to precisely control network topology not only provides avenues to engineer the properties of polymer gels but can also contribute to further unravel network structure–property relationships of these materials.

One interesting approach for the preparation of cross-linked polymers with well-defined network topologies involves the use of four-arm poly(ethylene glycol) (PEG) star macromers (Tetra-PEG).^14^ In seminal work, Sakai and co-workers demonstrated that cross end-coupling of two different macromers; viz., an *N*-hydroxysuccinimide ester (NHS) macromer terminated and an amine-end functional Tetra-PEG macromer afforded star copolymer networks, which are defect-free at length scales probed by small-angle neutron scattering and dynamic light scattering.^15^ By variation of the macromer concentration used in the cross-linking reaction, or by hydrolysis of a fraction of the NHS functional groups, this approach also allows to introduce defects into these networks in a controlled manner.^16^ In addition to the NHS-ester–amine coupling chemistry that was originally used by Sakai and co-workers, a number of other chemistries have also been successfully used to prepare networks from heterocomplementary reactive A4 and B4 type Tetra-PEG macromers. This includes Michael addition type chemistry using maleimide-end functional Tetra-PEG macromers and thiol- or amine-end functional Tetra-PEGs.^17−19^ Tetra-PEG gels have also been prepared via click reactions between azide-end functional Tetra-PEGs and dibenzocyclooctyne-, monofluorocyclooctyne-, and aryl-less cyclooctyne-modified Tetra-PEGs, or by reaction of norbornene- and 4-(6-methyl-1,2,4,5-tetrazine)phenylacetic acid-functionalized Tetra-PEGs.^20^ Other cross-linking chemistries that have been successfully employed to prepare Tetra-PEG gels involve the reaction between alkoxyamine- and ketone-end-functionalized building blocks^20^ and coupling of 2-(4-nitrophenyl)benzoxazinone and amine macromers.^21^

This paper reports the synthesis and structural characterization of disulfide-cross-linked Tetra-PEG networks that are obtained from self-reactive thiol-end functional Tetra-PEG macromers (Tetra-PEG-SH). The ability of thiol groups to self-react to form disulfide bonds is attractive, as it allows to investigate the formation and properties of Tetra-PEG networks generated via homopolymerization of A4-type macromers. So far, as mentioned above, Tetra-PEG networks are typically obtained via copolymerization cross-linking of equimolar quantities of heterocomplementary reactive A4 and B4 macromers. The formation of networks from self-reactive A4 macromers has been investigated in simulation studies.^22^ These simulation studies revealed that primary loops resulting from intramolecular cyclization are the predominant defect for networks prepared below the overlap concentration of the macromer. There are, however, only few cross-linking chemistries that would allow to experimentally validate the outcomes of these simulations. The oxidative cross-linking of Tetra-PEG-SH macromers represents an attractive model system to study the formation of polymer networks from self-reactive, A4-type-Tetra-PEG precursors. The resulting Tetra-PEG networks are also of interest, as the disulfide cross-links can be cleaved under reductive conditions, which makes these materials attractive as reduction-sensitive hydrogels that are relevant for a variety of biomedical applications.^23−27^ In this study, Tetra-PEG-SH macromers were homopolymerized under oxidative conditions in the presence of hydrogen peroxide to form disulfide-cross-linked networks. The structure of these networks was characterized by solid-state proton multiple-quantum NMR spectroscopy and small-angle neutron scattering experiments. These experiments indicated that the network architecture of disulfide-cross-linked Tetra-PEG networks, which were prepared at macromer concentrations that were 2.5 times larger than the overlap concentration, resembles that of the classical amide-cross-linked Tetra-PEG networks.

## Experimental Section

### Materials

All chemicals were used as received unless described otherwise. Four-arm-PEG thiol (Tetra-PEG-SH; *M*_n_ = 10 kDa and 90% SH substitution, as per the supplier) was purchased from Laysan Bio. Hydrogen peroxide (30%) was purchased from Reactolab SA. PBS tablets (pH = 7.4, NaCl = 140 mM, and KCl = 2.7 mM) were purchased from VWR International GmbH. Dithiothreitol (99%) was purchased from Fluorochem. Chloroform (HPLC grade) was purchased from Merck. Deuterium oxide was purchased from Sigma-Aldrich. Milli-Q water was obtained from a Millipore Direct-Q 5 ultrapure water system.

### Procedures

#### Preparation of Disulfide-Cross-Linked Tetra-PEG Gels

Disulfide-cross-linked Tetra-PEG gels were prepared at room temperature in PBS buffer (pH = 7.4) containing 0.2 v/v% H_2_O_2_. To prepare the gels, Tetra-PEG-SH macromer was dissolved in 0.2 v/v% H_2_O_2_ in PBS at the desired concentration (40, 60, 100, 150, or 200 mg/mL). The polymer solution was subsequently immediately cast into a Teflon mold containing 10 cylindrical wells with a diameter of 7 mm and a depth of 2 mm (∼ 100 μL polymer solution per well). The samples were allowed to cure at room temperature for 30 min. Samples for SANS and MQ-NMR experiments were prepared in 0.2 v/v% H_2_O_2_ in deuterium oxide (D_2_O) (instead of PBS). SANS samples were prepared in rectangular quartz cells (thickness: 1 mm, volume: 700 μL). Samples for MQ-NMR experiments were prepared in capped glass vials (volume = 1.5 mL; outer diameter = 11.6 mm; inner diameter = 6.2 mm; height = 32 mm) to prevent solvent evaporation.

#### Gel Fraction

To determine the gel fractions, the gels were dried overnight at 40 °C in a vacuum oven immediately after curing. For each gel, three replicates were weighed to obtain the initial dry weight (*m*_i_). The dried samples were subsequently immersed in water to leach out any remaining unreacted precursors. Water was changed every 3 h, and a total of three leaching cycles were performed. After that, the gels were dried in air overnight and then placed in a vacuum oven at 40 °C for 4 h to remove residual water. The washed and dried samples were weighed to afford the mass of the cross-linked fraction of the network (*m*_g_). From this, the gel fraction was calculated as
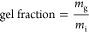
1

## Methods

### Small-Angle Neutron Scattering (SANS)

#### Data Acquisition and Processing

SANS experiments were carried out on the SANS-I instrument at the SINQ neutron source at the Paul Scherrer Institute (PSI), Villigen, Switzerland. The neutron wavelength was set to 8.00 Å with a wavelength spread of 10%. Sample-to-detector distances of 1.6, 4.5, and 18 m were used to cover the required *q* range. The scattered neutrons were counted with a 2D ^3^He-detector with 128 × 128 pixels of 7.5 × 7.5 mm^2^. The 2D detector data was calibrated using the incoherent scattering measured from a 1 mm-thick cuvette containing H_2_O, and the resulting data was converted to absolute units (1/cm). Taking sample transmissions into account, the raw data was corrected for dark background due to electronic noise and stray neutrons not originating from the sample as well as for sample background due to the solvent and scattering from the quartz-glass cuvette. The reduced 2D data were radially averaged after excluding the pixels blocked by the beamstop to obtain the macroscopic scattering cross section of the sample. This data reduction was carried out using the BerSANS software.^28^

#### Data Analysis

According to the c*-theorem of P.-G. de Gennes,^29^ the scattering function of a gel is equivalent to the scattering function of the precursor solution at the same concentration. For polymer solutions, the Ornstein–Zernike function^30^ is commonly used to describe the correlation length ξ of the system. As, however, polymeric systems are known to have a significant forward scattering contribution due to solid-like concentration fluctuations (e.g., chain associations or cross-linking inhomogeneities), a second term, characterizing a second length scale Ξ, is usually needed for a proper description. As has been shown^31−34^ on several types of gels (especially Tetra-PEG), the following equation usually provides an adequate description of the morphology of (inhomogeneous) gels:
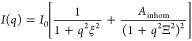
2

For simplification, the term *I*_0_ is used to capture scattering contrast, polymer volume fraction, temperature, and osmotic modulus, all of which will not be investigated in more detail in this study, as they do not contain structural information. The first term is the Ornstein–Zernike function that describes the length scale of liquid-like fluctuations ξ, which are commonly associated with the average network mesh size of a given network. The second term is a contribution according to Debye et al.,^35^ which characterizes the length scale Ξ of the solid-like fluctuations. The parameter *A*_inhom_ is introduced as an independent intensity contribution for this component.

### Solid-State Proton Multiple-Quantum NMR (MQ-NMR) Spectroscopy

Low-field MQ-NMR experiments were performed on a Bruker MiniSpec mq20 instrument operating at a magnetic field of *B*_0_ = 0.47 T and a temperature of 25 ± 2 °C. Pulse lengths were estimated to be between 1.5 and 2.0 μs for the 90° pulse and 3.0 to 4.0 μs for the 180° pulse. For each sample, a saturation recovery experiment, as well as the actual multi-quantum NMR (MQ-NMR) experiment, was performed. For the latter experiment, the Baum–Pines sequence^36^ with a recycle delay of 2 s was used. The short recycle delay was used to filter out most of the residual HDO signal, which would compete with the identification of isotropic defects. In the following paragraph, a short overview^37^ of the MQ-NMR methodology and the used fitting procedure will be given.

**Figure 1:**
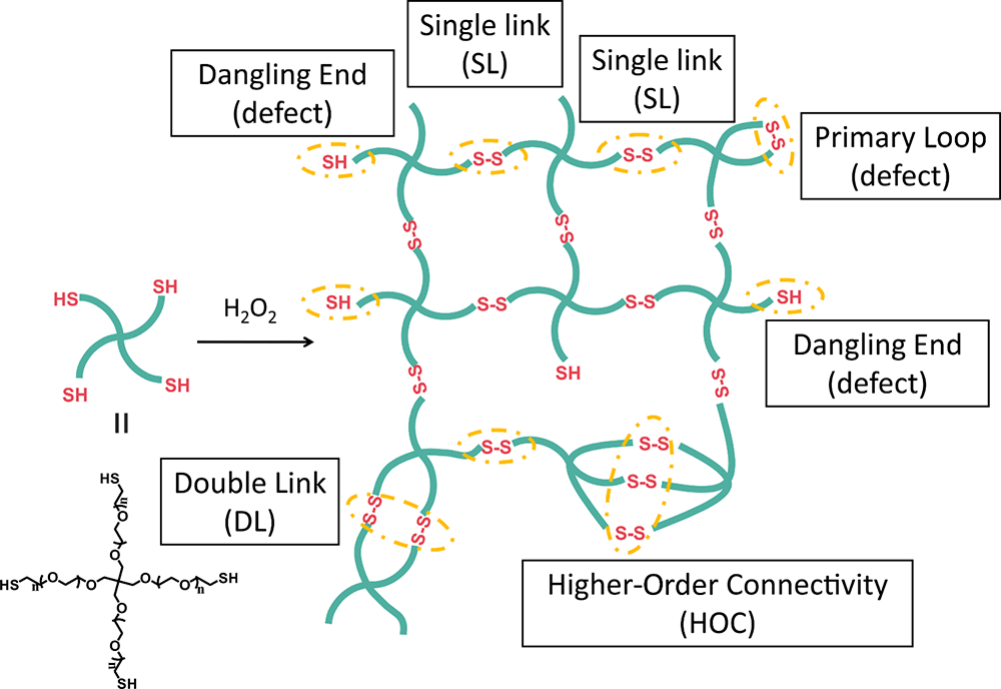
Preparation of disulfide-cross-linked networks from Tetra-PEG-SH macromers. The schematic illustration of the network structure highlights the main types of possible connectivity motifs, as well as defects that can be distinguished with MQ-NMR spectroscopy.

The Baum–Pines NMR sequence is used to obtain a set of two bulk-averaged time-domain signals, being on the one hand the double-quantum (DQ) buildup function (*I*_DQ_) and on the other hand a matching relaxation signal (*I*_ref_) in dependence of the double-quantum buildup time τ_DQ_. The former signal contains information about the bulk-averaged motional degree of freedom of all polymer backbone chains found in the sample, which is encoded in the form of motionally averaged residual dipolar coupling (RDC) that is directly accessed by the experiment. Broadly speaking, a chain that has no motional constraints and therefore is subject to isotropic reorientational motions will have a RDC value of zero whereas a sample that is subjected to strong motional constraints (e.g., cross-links, entanglements) will have a nonzero average between the dipolar couplings across the backbone protons, and therefore, a nonzero RDC value will be measured. As, however, the magnetization from the *I*_DQ_ signal is subject to relaxation, the resulting curve shape is a superposition of the DQ buildup and a decaying relaxation part, making it dependent on too many parameters for a simple regression procedure. Therefore, a relaxation-only signal is measured on top, which yields the possibility of fixing the “shape parameters” (relaxation times) and yielding a significant improvement of the fit quality. As initially shown by Lange et al.^38^ and refined in Bunk et al.,^21^ the following regression procedure can be used to extract quantitative information about the chain connectivity motifs (such as single links, double links, and higher-order connectivity motifs, as well as defects that can include primary loops and dangling ends; see Figure 1) in the Tetra-PEG networks:
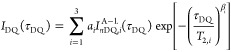
3
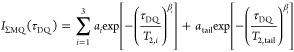
4Here, *I*_*n*DQ_ utilizes the Abragham-like function that was first derived in^39^ and is defined as
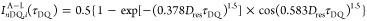
5

Normalized fractions of different connectivity motifs are decoded in the *a*_*i*_ prefactors, the respective RDC values are written as *D*_res,*i*_, and the corresponding relaxation times are written as *T*_2,*I*_. We will follow the notation from Lange et al.,^38^ where *a*_1_ corresponds to a single-link connectivity (SL), *a*_2_ corresponds to the double-link connectivity (DL), and *a*_3_ is used as an unspecified higher-order connectivity motif (HOC). Isotropic defects, such as dangling chain ends and sol, are found in the purely exponential long tail (*a*_tail_) of the relaxation curve (that does not have a corresponding DQ signal analogue). The empirical assignment is based on the idea that the measured dipolar couplings follow the simple relationship *D*_res_∼*M*_c_^–1^, as well as the simple idea that nonideal connectivities result in cross-links with a higher effective molecular weight. As in Tetra-PEG networks only a discrete distribution of connectivity motifs can occur, this assignment can be made using a top-to-bottom approach.

Both the full dipolar echo signal *I*_ΣMQ_ (= *I*_DQ_ + *I*_ref_) and the double-quantum buildup curve *I*_DQ_ are fitted simultaneously with eqs 3, 4, and 5 using a self-written regression procedure written in Python 3.10 and build on top of the *lmfit* library.^40^ In order to ensure a global minimum, the most significant parameter (the fraction of single links *a*_1_) is taken as a fixed value and systematically varied in small steps of Δ*a*_1_ = 0.01. The corresponding summed and scaled root-mean-squared errors (RMSE = RMSE_DQ_ + RMSE_ΣMQ_) are compared, and the global regression minimum is defined where RMSE shows a minimum. Error bars are defined empirically as the values where the RMSE surpasses a certain value (30% increase was chosen), resulting in a significant deviation that is easily seen by eye as an insufficient regression result. It should be noted that this approach yields rather precise values for *a*_1_ in sample series comparisons, but especially the ratio of *a*_2_ to *a*_3_ can be badly defined due to decreasing intensity with decreasing RDC value. Therefore, it is most useful to use the sum *a*_2_+*a*_3_ as a rather generic measure for the connectivity defects instead of discussing them separately.

### Rheology and Viscometry

Rheological and viscometric measurements were conducted using a TA DHR3 rheometer at 25 °C. For viscometric measurements, aqueous polymer solutions were prepared by dissolving Tetra-PEG-SH in Milli-Q water at concentrations ranging from 2.5 to 140 mg/mL. The specific viscosity was determined using a rheometer equipped with a 40 mm-diameter parallel plate geometry with a 0.5 mm gap. The critical overlap concentration c* was obtained from the plot between the specific viscosity (η_sp_) and the polymer concentration (*c*) as the crossover between the dilute and semidilute concentration regimes (Supporting Information Figure S1)^15,41^ and estimated to be 61.1 ± 6.6 mg/mL. Throughout this paper, for convenience, a c* of 60 mg/mL for the Tetra-PEG-SH macromers will be used.

To measure the storage and loss modulus of the Tetra-PEG gels, another set of experiments was performed by using a rheometer equipped with an 8 mm-diameter parallel plate geometry and a 1.0 mm gap. Rheological experiments were conducted on salt-free Tetra-PEG gels, which were obtained after three cycles of swelling in Milli-Q water (for 3 h). The desalted gels were dried overnight in a vacuum oven at 40 °C and then allowed to swell for 2 h in a Milli-Q water prior to analysis. Oscillatory frequency sweeps were conducted over a range of angular frequencies from 0.01 to 100 rad/s, while the strain was maintained at 1%.

## Results and Discussion

### Preparation of Tetra-PEG Gels

Disulfide-cross-linked Tetra-PEG gels were prepared as illustrated in Figure 1 from Tetra-PEG-SH macromers with a number-average molecular weight (*M*_n_) of 10 kDa. The Tetra-PEG gels were obtained from 0.2 v/v% H_2_O_2_ containing Tetra-PEG-SH macromer solutions in deuterium oxide (for NMR and SANS experiments) or in PBS (for rheology experiments). Under these conditions, gelation occurred within ∼2 min, which allowed transfer of the solutions into a mold. Increasing the H_2_O_2_ concentration accelerates the cross-linking kinetics. At a H_2_O_2_ concentration of 2%, for example, gelation is instantaneous. Gels were prepared from solutions containing 40–200 mg/mL Tetra-PEG-SH. To evaluate the cross-linking process, the gel fractions of Tetra-PEG networks prepared at different polymer concentrations were determined. Supporting Information Figure S2 presents the gel fractions that were determined for networks prepared in D_2_O and in PBS. Comparison of the gel fractions of networks synthesized in these two media does not reveal any significant differences, suggesting that the solvent does not appreciably influence the cross-linking process. The data presented in Supporting Information Figure S2, however, show that the gel fraction is strongly dependent on the macromer concentration that was used for the synthesis of the polymer networks. For Tetra-PEG networks prepared at a polymer concentration of 40 mg/mL, for example, gel fractions of 0.62 ± 0.08 and 0.69 ± 0.18 were determined for networks prepared in PBS, respectively, D_2_O. Increasing the polymer concentration used for the network synthesis to 60 mg/mL resulted in an increase of the gel fraction to 0.94 ± 0.03 (PBS) and 0.92 ± 0.04 (D_2_O). For networks prepared at macromer concentrations of 100, 150, and 200 mg/mL, the gel fraction further increased to 0.96 ± 0.03, 0.98 ± 0.01, and 0.99 ± 0.003 for networks prepared in PBS and to 0.97 ± 0.01, 0.98 ± 0.01, and 0.99 ± 0.01 for gels synthesized in D_2_O, respectively. The large increase in the gel fraction upon increasing the polymer concentration from 40 to 60 mg/mL coincides with the overlap concentration (c*) of the Tetra-PEG-SH macromer used in this study, which was estimated to be 60 mg/mL based on solution viscometry experiments (Supporting Information Figure S1). The determination of the gel fractions also indicates that for gels prepared at macromer concentrations that are 2.5 times higher than c* (i.e., ≥ 150 mg/mL), network formation is essentially quantitative as evident from the absence of a significant sol fraction.

### MQ-NMR Spectroscopy

**Figure 2:**
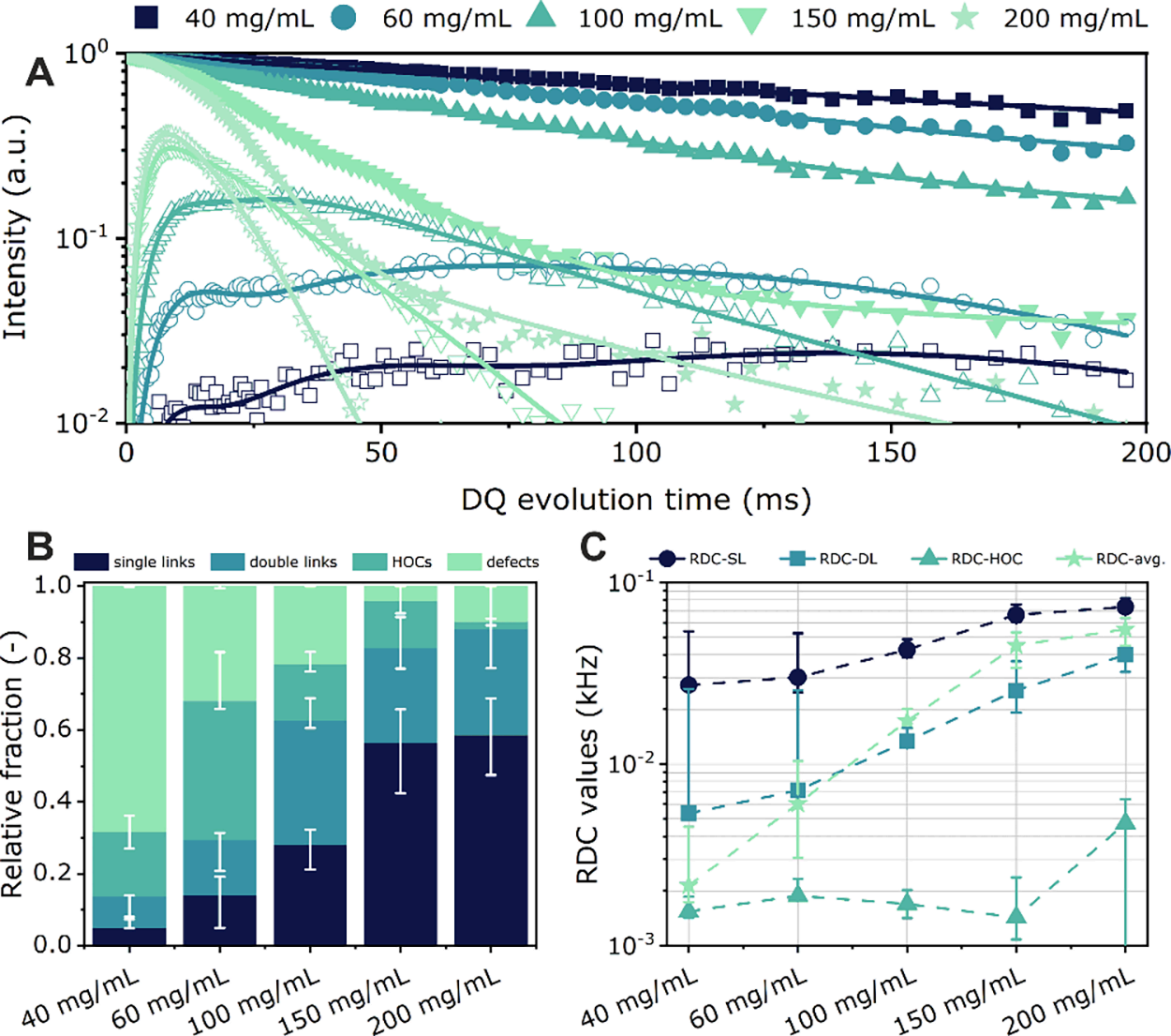
(A) *I*_ΣMQ_ (solid symbols) and *I*_DQ_ (open symbols) as measured by MQ-NMR spectroscopy for disulfide-cross-linked Tetra-PEG networks prepared with macromer concentrations ranging from 40 to 200 mg/mL. (B) Relative fractions of connectivity motifs, as well as (C) corresponding RDC values for disulfide-cross-linked Tetra-PEG gels obtained at different macromer concentrations.

Static time-domain MQ-NMR spectroscopy experiments were used to characterize the structure of the disulfide-cross-linked networks and to compare these materials with amide-cross-linked analogues obtained from NHS and amine functional Tetra-PEG macromers.^38^Figure 2A presents the experimental DQ buildup and MQ decay data that were obtained from the analysis of a series of disulfide-cross-linked gels, which were prepared at polymer concentrations ranging from 40 to 200 mg/mL. When the polymer concentration that is used to prepare the disulfide-cross-linked networks is increased, the MQ data display a decreasing tail, consistent with a decrease in isotropic defects. With increasing polymer concentration, the width of the DQ curve decreases, and its intensity increases, which indicates an increase in both the fraction and the RDC of the single-link component. While this behavior is expected for Tetra-PEG networks,^21,38,42^ the pronounced increase in the single-link fraction is surprising. Figure 2B and C presents the relative fractions of the different connectivity motifs and the corresponding RDC values, respectively. Figure 2B shows that disulfide-cross-linked gels prepared below the overlap concentration using a polymer concentration of 40 mg/mL (= 0.66 c*) only feature a very small fraction of single links (∼0.03–0.05) while the fraction of isotropic defects (∼0.70) is dominating, which suggests a fragile and poorly cross-linked network. This is attributed to the fact that in contrast to the heterocomplementary chain end-coupling reactions that are involved in the formation of conventional amide-cross-linked Tetra-PEG gels, the formation of the disulfide-cross-linked networks that is investigated here uses self-reactive Tetra-PEG-SH macromers. The formation of the disulfide cross-links is a nonselective end-linking reaction that also allows for first-order primary loops, where one arm of a star is connected to another arm of the same star. At higher concentrations, the spatial distance between end groups of different stars will decrease and the probability of interstar cross-links will increase. This is illustrated by the increase in the relative fraction of single links, and the decrease in the fraction of isotropic defects as the macromer concentration during network synthesis is increased from 40 to 200 mg/mL (Figure 2B). These observations confirm earlier simulation studies that highlighted the pronounced dependence of the different network connectivities, and especially of primary loop defects, on the polymer concentration for networks prepared by homopolymerization cross-linking of A4 macromers.^22^Figure 2C shows the RDC values for the different connectivity motifs, as well as the average RDC value, extracted from the fitting procedure. The concentration dependence of the RDC values of the DL and HOC components is more pronounced as compared to what has been observed on classical amide-cross-linked Tetra-PEG gels.^38^

**Figure 3:**
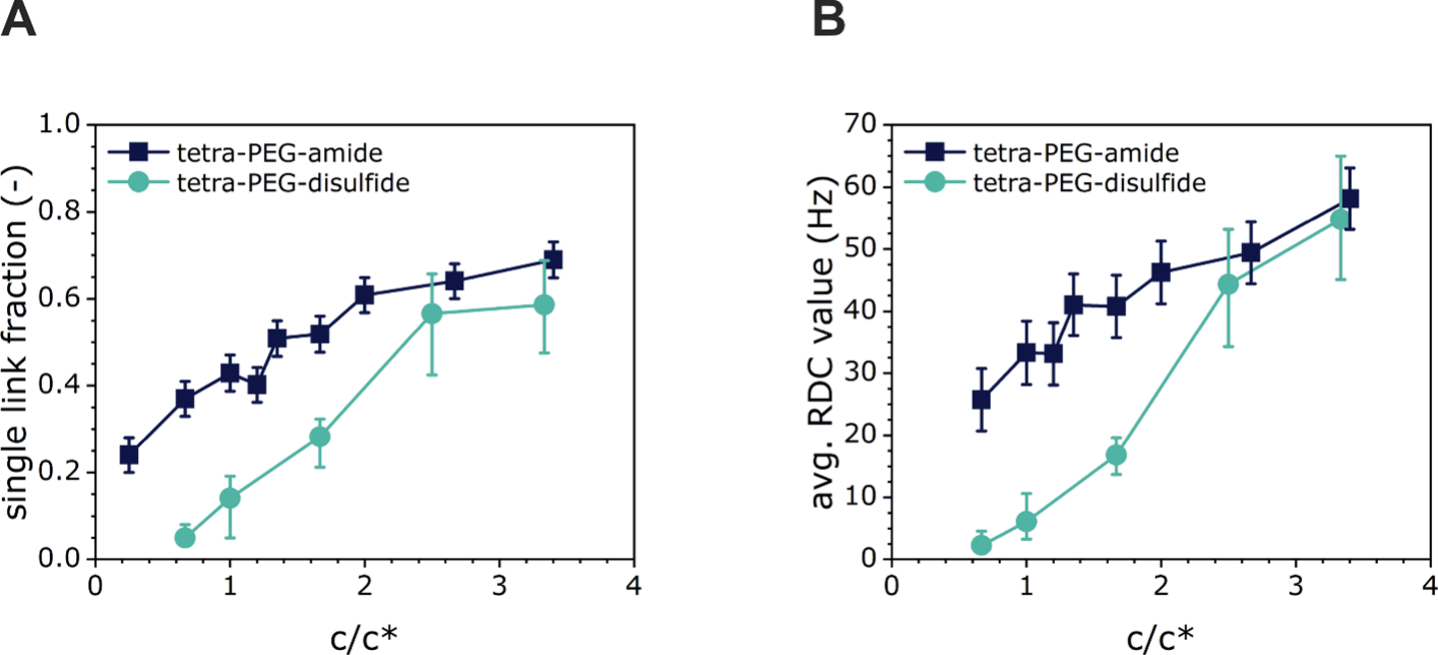
(A) The fraction of single links and (B) the average RDC value obtained from MQ-NMR spectroscopy analysis of the disulfide-cross-linked Tetra-PEG networks plotted together with the same data from amide-cross-linked Tetra-PEG gels (from ref (38)).

Figure 3 presents the single-link fraction (Figure 3A) and the average RDC values (Figure 3B) measured for the disulfide-cross-linked gels prepared at different polymer concentrations (plotted relative to the overlap concentration as *c*/c*) with the values obtained by Lange et al. on classical amide-cross-linked Tetra-PEG networks.^38^ For disulfide-cross-linked networks prepared at a Tetra-PEG macromer concentration of *c* = c*, a single-link fraction of 0.14 and an RDC of 9 Hz are found, as compared to a single-link fraction of 0.41 and an average RDC of 34 Hz for amide-cross-linked Tetra-PEG gels obtained at the same concentration. For gels prepared using Tetra-PEG macromer concentrations of *c* ≥ 2.5 c*, however, there is no significant difference neither with regard to the fraction of single links nor in terms of the average RDC between the disulfide-cross-linked Tetra-PEG networks and the classical amide-cross-linked tetra-PEG system. The lower average RDC values measured on the disulfide-cross-linked Tetra-PEG networks reflect the high content of primary loop defects in these gels obtained from self-reactive building blocks.

**Figure 4:**
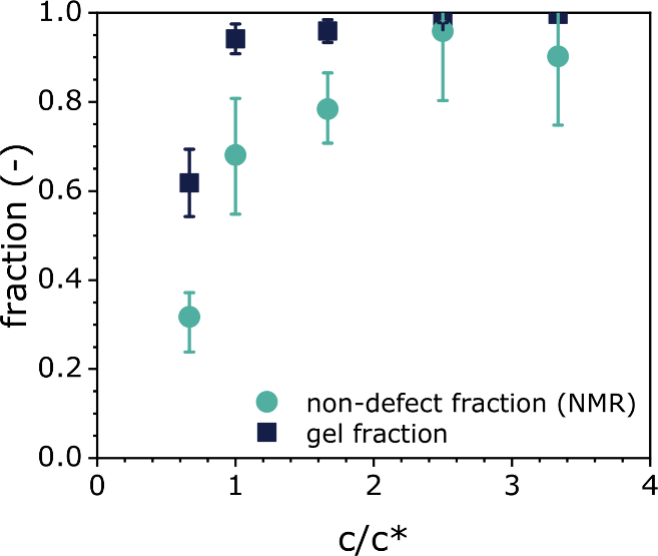
Non-defect fraction of network connectivities from MQ-NMR spectroscopy and gel fractions for disulfide-cross-linked Tetra-PEG networks synthesized at a range of macromer concentrations.

Figure 4 compares the non-defect fraction of connectivities as determined by MQ-NMR spectroscopy with the gel fraction for disulfide-cross-linked networks prepared at different Tetra-PEG-SH concentrations. For networks prepared at low macromer concentrations (*c* ≤ c*), there is a significant discrepancy between the non-defect fraction and the gel fraction, whereas this difference decreases for networks synthesized at higher Tetra-PEG-SH concentrations. The difference between gel fraction and non-defect content for networks prepared at macromer concentrations *c* ≤ c* is attributed to the presence of dangling chain ends, as well as primary loops. The formation of the latter is characteristic of networks obtained by cross-linking of self-reactive Tetra-PEG macromers. When the macromer concentration during network synthesis is increased, the difference between the gel fraction and the non-defect fraction decreases, which reflects the decrease in the number of primary loops in the network.

### SANS

**Figure 5:**
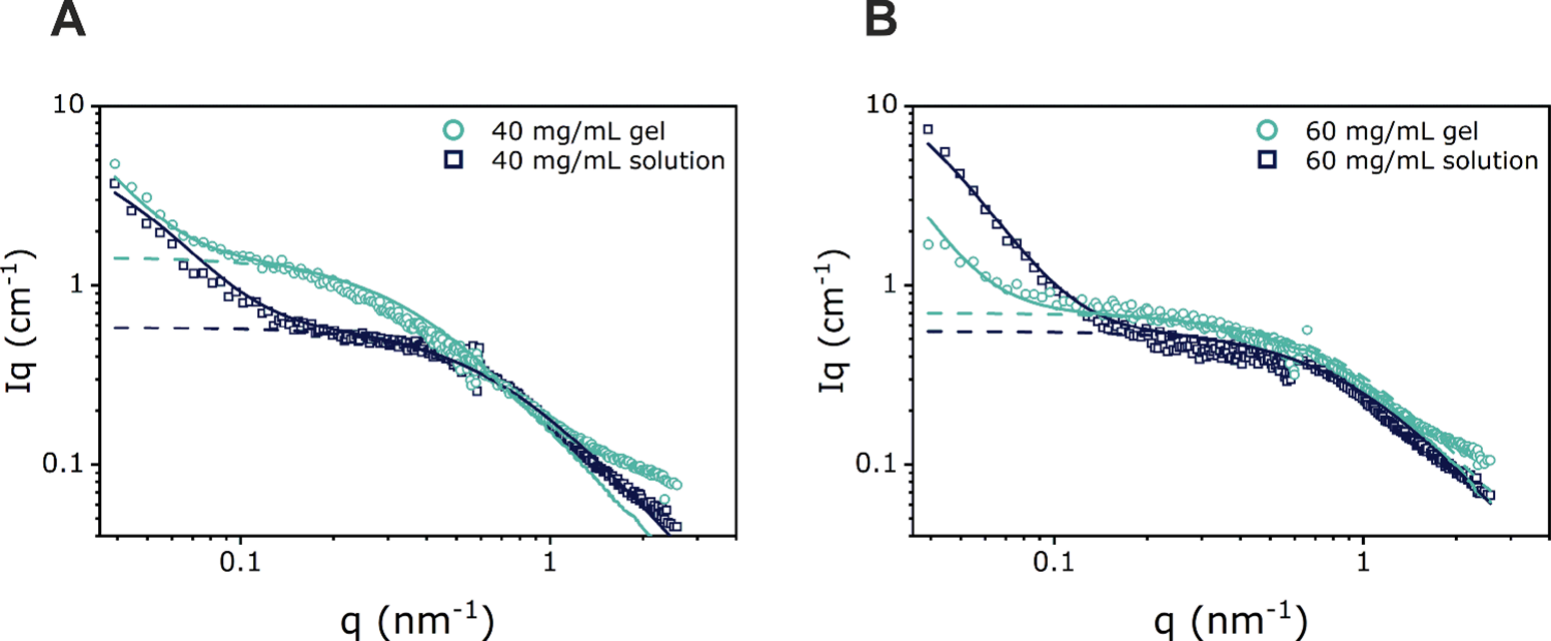
Scattering intensity curves of (A) a 40 mg/mL Tetra-PEG-SH macromer solution and a disulfide-cross-linked Tetra-PEG gel prepared at a macromer concentration of 40 mg/mL and (B) a 60 mg/mL Tetra-PEG-SH solution and a disulfide-cross-linked Tetra-PEG gel prepared at a macromer concentration of 60 mg/mL. The solid line represents the fit according to eq 2, whereas the dashed line shows the Ornstein–Zernike contribution.

To further probe the structure of the disulfide-cross-linked gels, SANS experiments were performed on networks prepared from Tetra-PEG-SH precursors at concentrations of 40 and 60 mg/mL. For comparison, SANS experiments were also performed on solutions of the Tetra-PEG-SH macromer at these two concentrations. Figure 5 presents the SANS curves obtained for the investigated disulfide-cross-linked Tetra-PEG networks and the corresponding Tetra-PEG-SH macromers.

For all samples, a strong upturn in scattering intensity at low *q* values is observed, as was also reported by Sakai et al.^33^ The scattering curves of both the macromer solutions and the disulfide-cross-linked networks consist of a clear superposition of two different contributions for liquid-like and solid-like concentration fluctuations that are modeled using eq 2 as a basis and which provides the correlation length ξ. From the c*-theorem, it is expected that the gel and the non-cross-linked precursor solution of the same concentration have a similar correlation length ξ. While this is satisfied (within the measurement error) for the network that was prepared at a macromer concentration of 60 mg/mL (i.e., *c* = c*) (solution: ξ = 1.5 nm, gel: ξ = 1.2 nm), a significant deviation is observed for the macromer solution and the polymer network obtained at 40 mg/mL (i.e., at *c* = 0.66c*) (solution: ξ = 1.6 nm, gel: ξ = 3.5 nm). The difference in correlation lengths ξ that are determined for the polymer network and the macromer solution at *c* = 0.66c* can be attributed to chain stretching that is required for the formation of cross-links at macromer concentrations that are lower than the overlap concentration. Due to the large fraction of defective connectivity motifs found by MQ-NMR spectroscopy, the measured correlation lengths do not correspond to the geometrical mesh size. A high number of isotropic defects (= dangling chain ends) will result in a highly defective network structure that will lead to the formation of higher-order loop structures,^43^ which by SANS are measured as the apparent mesh size ξ. SANS experiments on amide-cross-linked Tetra-PEG networks that were conducted by Sakai et al. indicated a similar dependence of ξ on the concentration of the Tetra-PEG macromer.^34^ From the correlation lengths of the networks that were obtained at concentrations of 40 and 60 mg/mL, an exponent of −1.6 can be estimated that describes the power law dependence of ξ on the macromer concentration. This exponent is larger as expected for a semidilute linear polymer in a good solvent (−0.75) but is comparable to the exponent determined by Sakai et al. for Tetra-PEG macromers with molecular weights of 5 and 10 kg/mol.^34^

### Rheology

**Figure 6:**
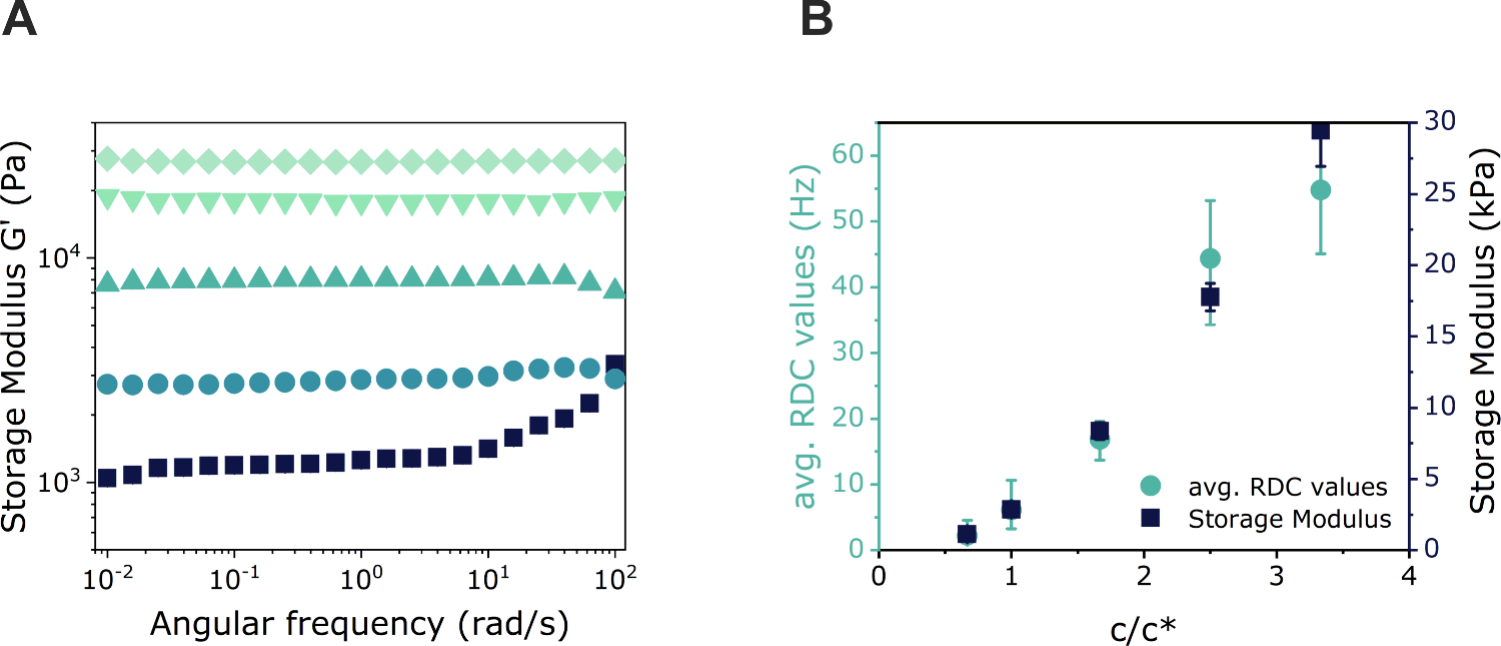
(A) Storage modulus *G*′ of disulfide-cross-linked Tetra-PEG gels prepared at polymer concentrations of 40 mg/mL (square), 60 mg/mL (circle), 100 mg/mL (triangle), 150 mg/mL (inverted triangle), and 200 mg/mL (diamond). (B) The weighted average of the measured RDC as obtained from MQ-NMR spectroscopy (left axis), as well as the plateau storage modulus *G*′ (right axis) from rheological measurements. In order to show the expected proportionality between these values, the RDC axis was scaled accordingly by definition of the upper limit value.

Rheological measurements were performed to study the viscoelastic properties of the disulfide-cross-linked gels. Supporting Information Figure S3 presents the angular frequency (ω) dependence of the storage modulus (*G*′) and loss modulus (*G*″) of Tetra-PEG gels prepared at macromer concentrations ranging from 40 to 200 mg/mL. For all gels, except those prepared at a macromer concentration of 40 mg/mL, *G*′ is ω-independent and *G*″ is at least two orders of magnitude smaller than *G′*. Figure 6A compares the frequency dependence of *G*′ for disulfide-cross-linked gels prepared at different Tetra-PEG-SH concentrations and shows a continuous increase in *G′* with increasing macromer concentration. The plateau values measured for *G′* on disulfide-cross-linked Tetra-PEG gels are similar to the values that have been reported for Tetra-PEG networks prepared using heterocomplementary cross-linking approaches at the same macromer concentration.^21,44,45^ In Figure 6B, the evolution of *G′* as obtained from rheology is plotted together with the average RDC value from the MQ-NMR analysis as a function of the macromer concentration used for the network synthesis. The similarities in the concentration dependence of these two parameters reflect the decrease in network defects as the polymer concentration increases.

## Conclusions

This study has used self-reactive, thiol-end functional Tetra-PEG macromers for the preparation of Tetra-PEG networks. Under oxidative conditions, homopolymerization cross-linking of these Tetra-PEG macromers affords disulfide-cross-linked polymer networks. The structure of the polymer networks was investigated by MQ-NMR spectroscopy and SANS. These experiments revealed a strong dependence of the network connectivities on the macromer concentration that was used to prepare the gels. At macromer concentrations of *c* = 0.66c*, networks were obtained that were predominantly composed of defect-type connectivites such as primary loops and dangling ends. For gels prepared at macromer concentrations *c* ≥ 2.5c*, the fraction of single-link connectivities in the disulfide-cross-linked gels was comparable to that in classical amide-cross-linked Tetra-PEG networks obtained by heterocomplementary cross-linking of *N*-hydroxysuccinimide ester and amine functional macromers. The pronounced concentration dependence of the network connectivities in these disulfide-cross-linked gels confirms findings from earlier simulation studies that investigated the formation of Tetra-PEG gels from self-reactive, A4-type, precursors. As disulfide bonds are susceptible to reductive cleavage, these disulfide-cross-linked gels are also of interest as reduction-sensitive hydrogels that are attractive for a variety of biomedical applications.

## Data Availability

The data generated and analyzed in this study have been uploaded to the Zenodo repository and are available at https://zenodo.org/records/10775482.

## Supporting Information Available

Solution viscometry of Tetra-PEG-SH, gel fractions of Tetra-PEG gels, and oscillatory shear rheology results (PDF)
