# Supplementary material for: Disulfide-Cross-Linked Tetra-PEG Gels
Source: Macromolecules. 2024 Mar 25;57(7):3058–65. doi: 10.1021/acs.macromol.3c02514 (PMC11008237; doi:10.1021/acs.macromol.3c02514)
Supplement: Supplementary file 1 — ma3c02514_si_001.pdf [file ma3c02514_si_001.pdf]

Supporting Information

Disulfide-Crosslinked Tetra-PEG Gels

*Zhao Meng,[1,2] Lucas Löser,[3] Kay Saalwächter,[3] Urs Gasser,[4] Harm-Anton Klok[1,2]**

[1] Institut des Matériaux and Institut des Sciences et Ingénierie Chimiques,

Laboratoire des Polymères, École Polytechnique Fédérale de Lausanne (EPFL),

Bâtiment MXD, Station 12, CH-1015, Lausanne, Switzerland.

[2] National Center of Competence in Research Bio-inspired Materials.

[3] Institut für Physik - NMR, Martin-Luther Universität Halle-Wittenberg,

Betty-Heimann-Str. 7, 06120 Halle (Saale), Germany.

[4] Laboratory for Neutron Scattering and Imaging (LNS),

Paul Scherrer Institut, CH-5232 Villigen PSI, Switzerland.

* Corresponding author

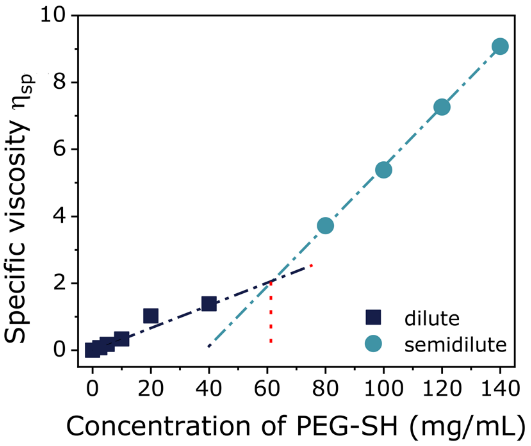

Figure S1: Specific viscosity (η_{sp}) of aqueous Tetra-PEG-SH macromer solutions with polymer concentrations ranging from 2.5 – 140 mg/mL. The intersection of the linear fits of the lower concentration (dilute) and higher concentration (semidilute) regime was taken as the overlap concentration c*.

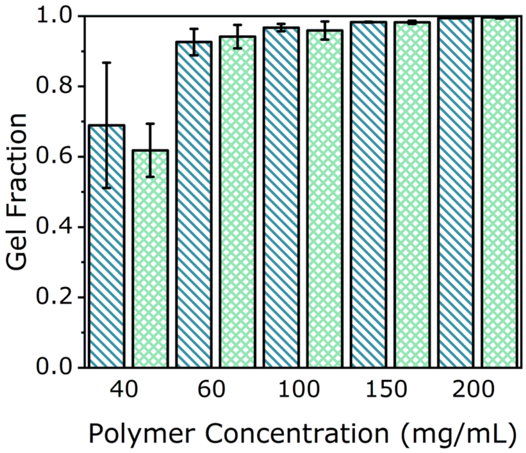

Figure S2: Gel fractions of disulfide-crosslinked Tetra-PEG gels prepared with polymer concentrations ranging from 40 mg/mL to 200 mg/mL in D_2O (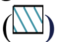) and in PBS (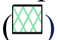) as the solvent.

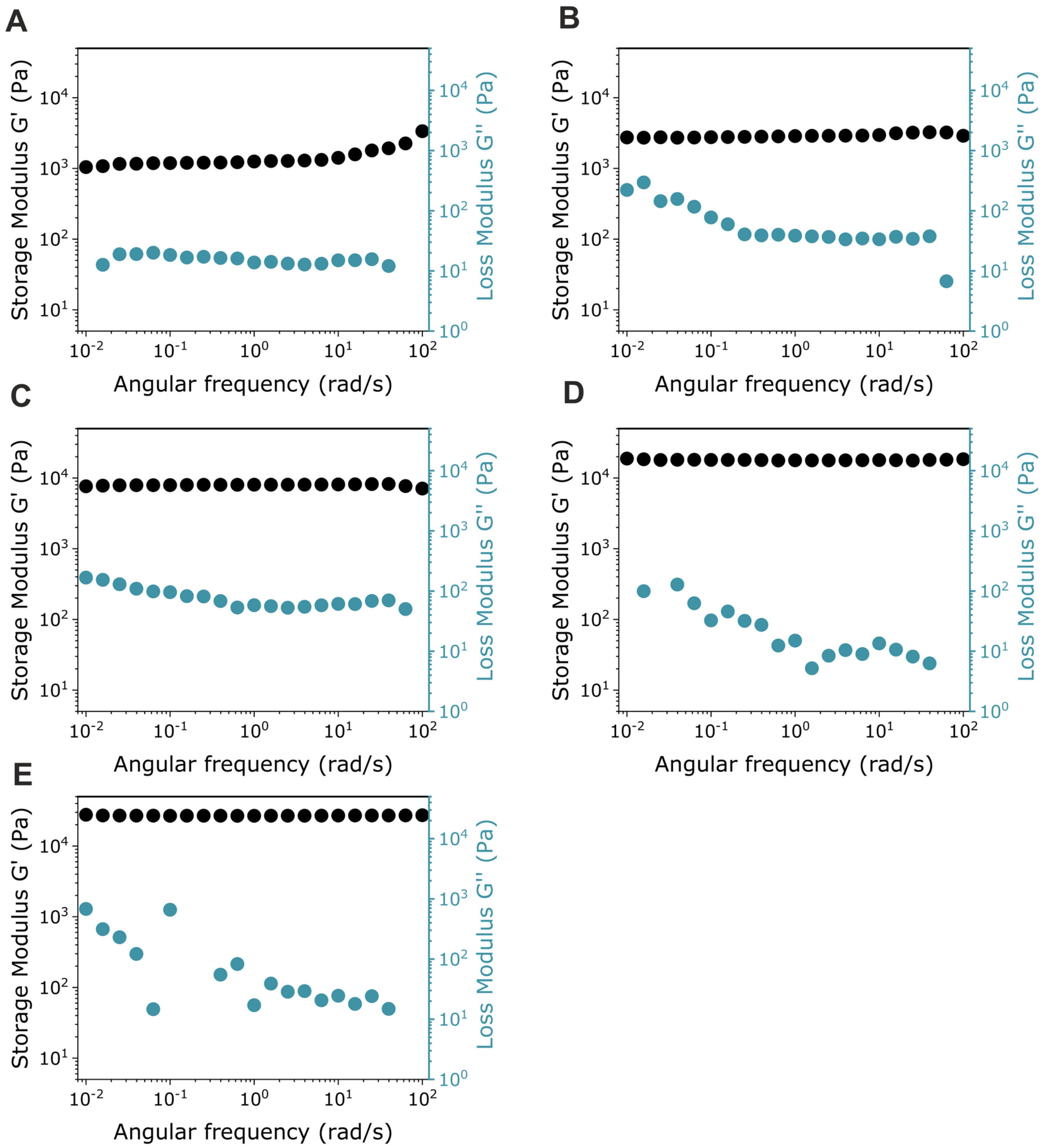

Figure S3: Oscillatory shear-rheology of disulfide-crosslinked tetra-PEG gels prepared at a polymer concentration of (A) 40 mg/mL, (B) 60 mg/mL, (C) 100 mg/mL, (D) 150 mg/mL, and (E) 200 mg/mL.
